# Selective Extraction of Valuable and Critical Metals in Cassiterite Concentrate by Dry Chlorination, Part I: Thermodynamic and Modelling Perspective

**DOI:** 10.3390/ma17174186

**Published:** 2024-08-23

**Authors:** Allen Yushark Fosu, Bastien Demeusy, Frédéric Diot, Tiina Lavonen, Veronika Meriläinen, Danièle Bartier, Yann Foucaud, Ndue Kanari

**Affiliations:** 1 GeoRessources, CNRS, Université de Lorraine, F-54000 Nancy, France; bastien.demeusy@univ-lorraine.fr (B.D.); frederic.diot@univ-lorraine.fr (F.D.); daniele.bartier@univ-lorraine.fr (D.B.); ndue.kanari@univ-lorraine.fr (N.K.); 2 VTT Technical Research Centre of Finland, FI-02044 Espoo, Finland; tiina.lavonen@vtt.fi (T.L.); veronika.merilainen@vtt.fi (V.M.)

**Keywords:** tin concentrate, critical and strategic metals, chlorination, thermodynamic modelling, extraction, selective separation

## Abstract

The chlorination of oxides of major concern in cassiterite concentrate with various chlorinating agents is investigated in light of their thermodynamic feasibilities to extract and recover their valuable metal components. Mechanisms responsible for the processes and their Gibbs free energy changes as a function of temperature to selectively separate and/or recover the metal(s) of interest and unwanted ones as their metallic chlorides are identified. Attention is given to gaseous (Cl_2_ and Cl_2_ + CO mixture) and solid (CaCl_2_ and MgCl_2_) chlorine sources, from which Cl_2_ + CO shows no reaction selectivity for any of the oxides but a feasible metal chloride formation for all. Chlorine gas (Cl_2_), on the other hand, could selectively form chlorides with metals of +2 oxidation state in their oxides, leaving those of high oxidation state unreacted. MgCl_2_, unlike CaCl_2_, is found capable of producing calcium, ferrous, and stannic chloride from their metallic oxides with enhanced reaction tendencies in the presence of silicon dioxide (SiO_2_). An overall study of the thermodynamic feasibility of all chlorine sources looked at alongside operational and environmental viabilities suitably suggests MgCl_2_ for a selective extraction of the valuable metal components in a cassiterite concentrate, in which case, moderate temperatures seem promising.

## 1. Introduction

**Figure 1:**
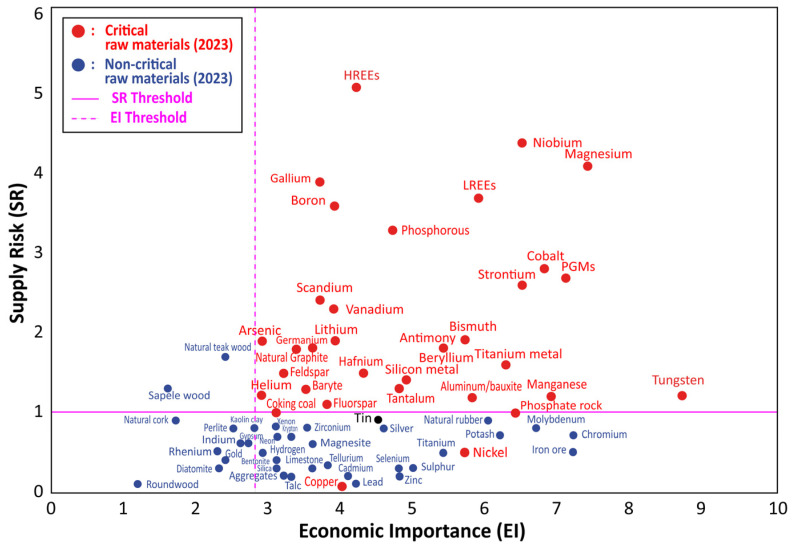
Correlation between economic importance and supply risk of raw materials for the European Union in 2023.

Though the importance of metals, in general, in the livelihood of mankind cannot be overemphasized, a couple of them, due to their crucial significance, standout, catching the attention of policy makers, stakeholders, and metallurgists worldwide. The same can be said of some metal-containing substances. They have been termed “Critical and Strategic Raw Materials” (CRMs). There has not been a clear cut or globally accepted definition for these materials as it is subjective to a country’s need regarding its resource endowment and production capacity, national and economic security, technological evolution, and risk of supply [1]. Thus, metals that may be classified as critical and strategic by one country may not necessarily be the same for another. Critical metals may be used interchangeably to mean strategic metals; however, both may have different connotations. Critical metals may refer to those metals that are indispensable for the green technology transition but have a specific geographic deposit and production, making it vulnerable and risky in their supply due to monopoly [1,2]. Strategic metals, on the other hand, have economic, health, and military inclination [1,2]. That is, those metals that are used for medical, defense, and security equipment as well as the economic development of a country [1]. Figure 1 shows materials that are regarded as critical and strategic for the European Union, indicating their supply risk and economic importance [3].

Niobium (Nb) and tantalum (Ta) fall in the European Union (EU) CRMs category. They occur as accessory material with cassiterite (SnO_2_). Both are d-block transition metals with similar characteristics often referred to as “geochemical twins” due to the closeness of their mass-to-charge ratio [4,5]. Their twin-like nature resulted in severe controversy during their early stage of isolation, identification, and naming such that they were mistakenly thought to be one and the same element. A common name (tantalum) was attributed to this supposed same metal until a detailed investigation revealed them to be different but similar, naming the lighter element “niobium” to differentiate it from the heavier one (tantalum). They are both group 5 elements, slightly acidic, body-centered, cubic, and refractory, with electronic configurations of [Kr]4d^4^5s^1^ and [Xe]4f^14^5d^3^6s^2^, respectively, giving them a +5 maximum oxidation state [6].

Generally, cassiterite is sourced either from placer (alluvial) or hard rock (granite/pegmatite) deposits, wherein the latter hosts the metals and remain in the concentrate after processing. The cassiterite-containing ore goes through severe enrichment processes to obtain the concentrate, which sometimes leads to low recovery of niobium and tantalum. Composition of the mineral concentrate or the ore from different locations, as indicated by some researchers [7,8,9,10] is shown in Table 1.

**Figure 2:**
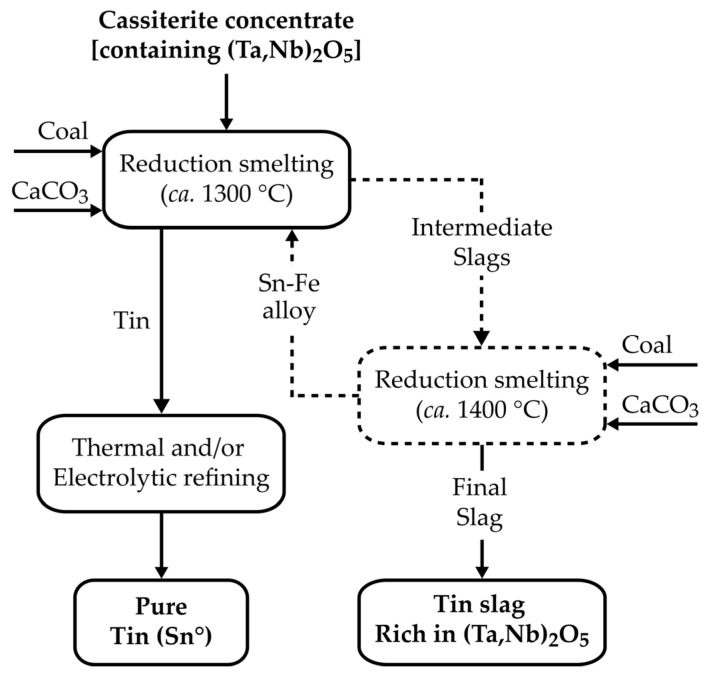
Simplified flowsheet for the treatment of the cassiterite concentrate by pyro-metallurgical route.

CRMs in cassiterite are usually recovered from the slag obtained after extracting the tin content using the carbothermal reductive smelting method (Figure 2). The smelting is a two-step energy-intensive process (usually operated around 1300 °C and 1400 °C, respectively, for the first and second stage) before the slag rich in CRMs is obtained [11].

Another extractive pathway for recovering the metallic components from cassiterite that has, however, received little attention is dry chlorination. This is a thermal process where a suitable chlorine source (either gas or solid) is made to react with the mineral or material containing the metal of interest at a specific temperature or temperature range. This technique is a chlorine–oxygen exchange reaction that leads to the formation of a chloride or oxychloride of the metal for downstream processing. The technique has been explored to extract metals from both primary and secondary sources [12,13,14,15,16,17]. In this regard, the extraction of several metals, including potassium, lithium, gold, etc., from feldspars [18,19], spodumene or lepidolite [20,21], and alluvial gold-containing material [22] as their primary sources are typical examples, whilst the processing and recovery of the valuable components of slags and solid wastes (as secondary sources) are known [23,24]. It operates at comparatively lower temperatures than other pyrometallurgical methods and is very effective for sources of metals whose grade is low and/or is recalcitrant to extraction toward other extractive routes [12]. For instance, α-spodumene, which is resistant to chemical attack, has successfully been treated by this technique to extract lithium at a relatively lower temperature than what would be expected in the tradition approach [12]. The approach is even more successful, requiring further lower operating temperatures if conducted in the presence of a reductant or reducing environment than without it [25,26,27,28]. The importance of reductant or a reducing atmosphere to the technique is confirmed by Refs. [29] and [26] during their study on MgO and tin slag, respectively. The former recorded an increasing reaction order of 0.89, 1.47, and 2.37 with respect to CO, Cl_2_, and Cl_2_ + CO with about 200 °C lower reaction initiation temperature for the carbochlorination than chlorination. The later also documented a similar observation for the extraction of niobium and tantalum from a high-grade tin slag sample.

Gaseous, liquid, or solid sources of chlorine have been employed to concentrate, open up, extract, and refine some raw materials or as a synthesis route for some useful products, with evidence of its use as a remediation technology also known. The dry chlorination technique uses either the gaseous (specifically Cl_2(g)_ and, to a lesser extent, HCl_(g)_) or the solid form (such as MgCl_2_, CaCl_2_, NH_4_Cl, AlCl_3_, etc.) of chlorine. The gaseous reagents benefit from easy diffusion through the sample, enabling fast interaction with the target atoms, which enhances the reaction rate. The use of gaseous reagents, therefore, favors the process kinetics more than their solid counterparts, yet their corrosive and toxic nature coupled with their handling difficulty, especially during transportation, is a demerit to the process application. Corrosion-resistant equipment is required for their set-up, which makes it complicated and expensive to implement. Dry chlorination application to niobium and tantalum extraction from several sources and chlorination agents in the presence or absence of reducing agents have been documented [26,30,31,32,33].

Mineral types (oxidic (MO), sulphide (MS), or silicate (MO·SiO_2_); where M is a metal) respond differently during chlorine metallurgy and may or may not require additives alongside the chloride reagent to enhance the metal extraction. For instance, minerals (such as oxidic minerals and silicates) with low amenability to direct chlorination may require a carbon-source reducing agent (especially carbon or carbon monoxide) to enhance the system’s potential activity and promote the formation of the respective metal chloride [25,34,35]. In that case, the process is termed carbochlorination. Some ores (especially the sulphides), on the other hand, may require an oxidizing agent to enhance the process feasibility in which the term oxychlorination is used [25]. Thus, the choice of a reagent and additive suitable for a metal’s extraction from a mineral by chlorine metallurgy plays an important role for the process feasibility. The higher reactivity of carbochlorination (Cl_2_ + CO mixture) over the chlorination (Cl_2_) counterpart has been documented by Brocchi et al. [30], whilst the greater efficiency of oxychlorination (Cl_2_ + O_2_) than its chlorination (Cl_2_) counterpart was confirmed by Gaballah and coworkers during their CrO_2_Cl_2_ production from Cr_2_O_3_ [35].

With the depletion of high-grade ore and much attention geared toward lean grades, the application of chlorine metallurgy becomes complicated since, in most cases, formation of refractory new phases that do not respond well to the technique occurs. Sometimes, there may be reduced metal recovery resulting from passivation or occlusion of the metal of interest because of the formation of new recalcitrant phases. An earlier researcher [36] also investigated the effect of some gangue (MgO, SiO_2_, Al_2_O_3_, CaO, K_2_CO_3_) on the reduction smelting of cassiterite using carbon, where a catalytic effect on the Boudouard reaction was exhibited by most of the materials and led to reduced activation energy of the entire process. This study hereby aims to investigate thermodynamically, the chlorination and carbochlorination behaviors of some metal oxides in cassiterite concentrate whose presence may impact the extraction of its valuable metal content. Oxides that occur in appreciable amounts in the concentrate or ore (Table 1) were considered in the study. Critical and strategic niobium and tantalum pentoxides will be considered exclusive in the part 2 of this study. Gaseous reagents (particularly Cl_2_ and Cl_2_ + CO mixtures) are explored in detail, but, due to the challenges it presents, the study is extended to include solid reagents (CaCl_2_ and MgCl_2_) to evaluate how best the two compare for the process efficiency. Knowledge of this will help to predict the possible separation and refining of valuable metal and/or groups of valuable metals from gangues in a cassiterite concentrate.

Thermochemical softwares are useful tools that enables process engineers to simulate processes and chemical reactions, giving useful information before experimentation. It helps to predict which processes are likely or unlikely to occur in real life alongside giving useful suggestions on the process conditions and possible products to expect. These are handy information that prevents ad hock and trial-and-error experimental tests.

HSC Chemistry Software^®^ version 5.1 (Outokumpu Research, Oy, Finland), like other thermochemical software, has been used by several researchers [12,37,38,39] to envisage process feasibilities and their corresponding condition and possible expected products as well as give clues to explain some experimental results. Reference [12], with the help of this tool, selected CaCl_2_ among a series of reagents as the suitable one capable of extracting lithium directly from the α-form of spodumene whilst revealing anorthite and lithium chloride as the main end products. These revelations from the simulation were later confirmed experimentally in their study. In the same study, the software was used to satisfactorily predict the spodumene phase transformation initiation temperature, which has been proven in an earlier study. In a similar study, Barbosa et al. [21,40] as well as Rosales et al. [41] used the equilibrium module of the software to envisage the reaction conditions and possible expected products for the β-spodumene reaction with calcium chloride, chlorine gas, and sodium fluoride, which were in good agreement with experimental findings. Wang and his colleagues [39] have unraveled the possible conditions and mechanism responsible for the decomposition of zinc ferrite (ZnFe_2_O_4_) to zinc oxide, hematite, or wustite using a combination of both the reaction equation and equilibrium module of the software. The chlorination, carbochlorination, and oxychlorination behaviors of a number of minerals have been simulated prior to experimental tests. In most of these cases, experimental results seem to confirm the simulation outcomes [40,41].

Though the software gives important and reliable information on processes, Pickles and Marzoughi [42] highlighted some shortfalls that must be considered by the operator. First, a closed system is assumed by the software; thus, it considers that no specy (reactant or product) is lost in the process, and if gases are generated, they remain and interact with the system, but this does not always happen in real life. Also, the software depends on data stored in it. The correctness of calculations therefore depends on the accuracy of the database, though the operator has the free will of adding on data that is thought to be accurate and reliable. Also, the software does not take the kinetics of processes into account. Due to the associated shortfalls of the software, they made an attempt to evaluate the modeling results of an adjusted database of the software through the addition of available literature with thermodynamic data of lithium aluminosilicates in order to mimic the decrepitation behavior of both spodumene concentrate and pure spodumene. Like other revelations by the software, the modeled decrepitation results agreed very well with data encountered in the literature. In a nutshell, these previous studies validate the outcomes of the software and, hence, its reliability of the findings in the current investigation.

Generally, the software predicts process feasibilities using the reaction equation module of the software and data stored in it to calculate the standard Gibbs free energy changes of the reaction(s) governing the process within the temperature range under investigation. The equilibrium composition of the reaction is also predicted using the equilibrium module of the software with a plot of equilibrium composition as a function of temperature as the output. Readers are directed to the “Equilibrium calculations” section of Ref. [42] for detailed information on how this module of the software is operated to achieve reliable results.

Process feasibilities are determined using the arithmetic sign (negative or positive) of the standard Gibbs free energy (${\Delta G}^{o}$) change of the reaction, which is obtained by the expression ${\Delta G}^{o}$ = ${\Delta H}^{o}$ −T${\Delta S}^{o}$, where ${\Delta H}^{o}$, T, and ${\Delta S}^{o}$ are the standard enthalpy change, absolute temperature, and standard entropy change, respectively. The software has, in its database, a collection of data for a plethora of chemical species that may be displayed in the absolute or the delta form depending on the command issued by the user. The user, however, has the option of adding into the software, a personal data of chemical species (if reliable), which are stored in the “Own database” and given a priority during calculation over the “Main database” of the software. To explore the feasibility of a process, the reaction equation module is selected and the chemical reaction governing the process is written in the reaction equation box, taking into consideration the physical states of the species. The “Balance Equation” option may be of help to obtain the stoichiometric coefficient of the species. The temperature range required for the process is then specified, after which the software calculates and displays the calculated ${\Delta H}^{o}$, ${\Delta S}^{o}$, and, hence, ${\Delta G}^{o}$ for the specified temperature range using information from the database stored in it. The software assumes that all species in the reaction are in their standard states, hence ${\Delta G}^{o}$ may not be the actual determinant of a process feasibility but the free energy change of the reaction (ΔG), which is expressed as: ΔG = ${\Delta G}^{o}$ + RTln K, where R and K are the universal gas and equilibrium constants, respectively. Despite this observation, ${\Delta G}^{o}$ has generally been used by process engineers for the estimation of process feasibilities.

## 2. Chlorination and Carbochlorination of Metallic Oxides in Cassiterite Concentrate Using Reactive Gases

**Figure 3:**
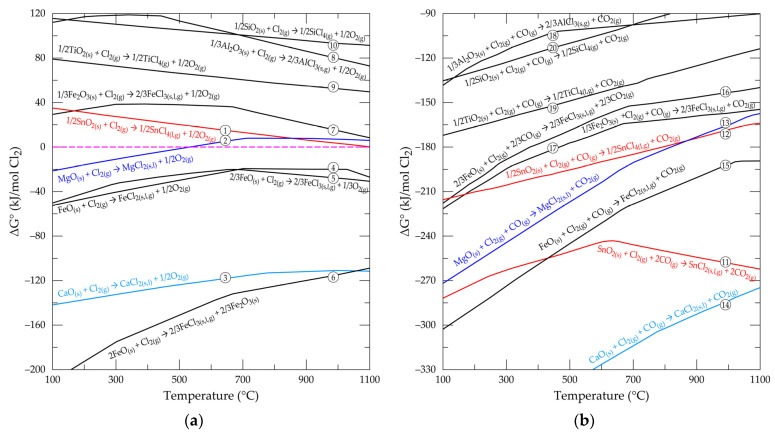
Evolution of standard free energy changes as function of temperature for the reactions of selected oxides with (**a**) Cl_2_ and (**b**) Cl_2_ + CO.

Figure 3a,b is the plot of Gibbs free energy changes (${\Delta G}^{o}$) as a function of temperature for the chlorination and carbochlorination (Equations (1)–(20)) of major oxides in cassiterite concentrate using Cl_2_ and Cl_2_ + CO gas mixtures, respectively. To facilitate easy understanding, all equation numbers have been indicated on their respective curves as well. Curves of divalent metal chlorides (SnCl_2_, MgCl_2_, CaCl_2_, and FeCl_2_) in the graphs are characterized by changing slopes that correspond to the physical state transformation of the chlorides. For instance, curves of SnCl_2_ and FeCl_2_ have changing slopes around 246 and 677 °C, respectively, which correspond to their solid-to-liquid transformation, and another occurring at about 623 °C and 1023 °C, respectively, also corresponding to their liquid-to-gas transformation. The higher oxidation state (+4) chlorides (SnCl_4_, TiCl_4_, and SiCl_4_), which appear to be gases at low temperatures, do not mostly show any appreciable change of slope in their curves.

1/2SnO_2(s)_ + Cl_2(g)_ → 1/2SnCl_4(g)_ + 1/2O_2(g)_ (1)

MgO_(s)_ + Cl_2(g)_ → MgCl_2(s,l)_ + 1/2O_2(g)_ (2)

CaO_(s)_ + Cl_2(g)_ → CaCl_2(s,l)_ + 1/2O_2(g)_ (3)

FeO_(s)_ + Cl_2(g)_ → FeCl_2(s,l,g)_ + 1/2O_2(g)_ (4)

2/3FeO_(s)_ + Cl_2(g)_ → 2/3FeCl_3(s,l,g)_ + 1/3O_2(g)_ (5)

2FeO_(s)_ + Cl_2(g)_ → 2/3FeCl_3(s,l,g)_ + 2/3Fe_2_O_3(s)_ (6)

1/3Fe_2_O_3(s)_ + Cl_2(g)_ → 2/3FeCl_3(s,l,g)_ + 1/2O_2(g)_ (7)

1/3Al_2_O_3(s)_ + Cl_2(g)_ → 2/3AlCl_3(s,g)_ + 1/2O_2(g)_ (8)

1/2TiO_2(s)_ + Cl_2(g)_ → 1/2TiCl_4(g)_ + 1/2O_2(g)_ (9)

1/2SiO_2(s)_ + Cl_2(g)_ → 1/2SiCl_4(g)_ + 1/2O_2(g)_ (10)

SnO_2(s)_ + Cl_2(g)_ + 2CO_(g)_ → SnCl_2(s,l,g)_ + 2CO_2(g)_ (11)

1/2SnO_2(s)_ + Cl_2(g)_ + CO_(g)_ → 1/2SnCl_4(g)_ + CO_2(g)_ (12)

MgO_(s)_ + Cl_2(g)_ + CO_(g)_ → MgCl_2(s,l)_ + CO_2(g)_ (13)

CaO_(s)_ + Cl_2(g)_ + CO_(g)_ → CaCl_2(s,l)_ + CO_2(g)_ (14)

FeO_(s)_ + Cl_2(g)_ + CO_(g)_ → FeCl_2(s,l,g)_ + CO_2(g)_ (15)

2/3FeO_(s)_ + Cl_2(g)_ + 2/3CO_(g)_ → 2/3FeCl_3(s,l,g)_ + 2/3CO_2(g)_ (16)

1/3Fe_2_O_3(s)_ + Cl_2(g)_ + CO_(g)_ → 2/3FeCl_3(s,l,g)_ + CO_2(g)_ (17)

1/3Al_2_O_3(s)_ + Cl_2(g)_ + CO_(g)_ → 2/3AlCl_3(s,g)_ + CO_2(g)_ (18)

1/2TiO_2(s)_ + Cl_2(g)_ + CO_(g)_ → 1/2TiCl_4(g)_ + CO_2(g)_ (19)

1/2SiO_2(s)_ + Cl_2(g)_ + CO_(g)_ → 1/2SiCl_4(g)_ + CO_2(g)_ (20)

Chlorination reactions with Cl_2_ may form the chlorides or oxychlorides of the respective metal; nonetheless, the metal chloride products are considered in this study. It is observed that the inclusion of carbon monoxide in the gas mixture has a profound effect on the ${\Delta G}^{o}$ value and, hence, the feasibility of the reactions. The heating of the carbon monoxide and chlorine gas mixture creates equilibrium between them and phosgene (COCl_2_) as shown in Equation (21) to initiate the reaction, which progresses in the following four stages: (1) chemisorption of Cl_2_ on the sample surface, (2) formation of a stimulated phosgene coordinated layer, (3) metal oxide–phosgene complex interaction and product formation, and (4) desorption of products and entrainment into the off-gas [43,44]. Thus, during the process, chlorine gas is first chemisorbed on the surface of the metal oxide, and subsequently by the carbon monoxide to form the phosgene–metal complex, which sparks the reaction for the release of carbon dioxide and the metal chloride [45,46]. Even in cases where carbon instead of carbon monoxide is used, the above mechanism is partially followed as the process progresses through in situ generation of CO by the metal oxide–carbon reaction with Cl_2_ and the Boudouard reaction (Equations (22) and (24)) for the metal chloride formation [45,47]. It can therefore be said that two mechanisms come into play for the formation of the metal chlorides from their oxides, one through the Cl_2_/C reaction (Equation (22)) and the other by Cl_2_/CO (Equation (23)), where an equal contribution for the bulk metal chloride synthesis has been indicated [47].

CO_(s)_ + Cl_2(g)_ → COCl_2(g)_ (21)

MO_2(s)_ + 2Cl_2(g)_ + 2C_(s)_ → MCl_4(s,l,g)_ + 2CO_(g)_ (22)

MO_2(g)_ + 2CO_(g)_ + 2Cl_2(g)_ → MCl_4(s,l,g)_ + 2CO_2(g)_ (23)

C_(s)_ + CO_2(g)_ → 2CO_(g)_ (24)

In some cases, small quantities of products can be achieved from some dry chlorination reactions in real life, despite their thermodynamic impossibility. This speculation is attributed to the interaction of the chlorine and oxygen atom of the metal oxide at some sites, particularly the corners and edges at the crystal surface of the sample. Generally, interfacial oxygen and those found at the aforementioned strategic positions possess unduly high energy due to the incomplete bonding of atoms at these locations in the sample. There is, therefore, some sort of “partial feasibility reaction” leading to product formation regardless of their unfavorable thermodynamic conclusion. As this reaction is an interfacial phenomenon rather than of the bulk sample, resulting products are usually minimal. Equation (8) has a very high positive ${\Delta G}^{o}$ value but Ref. [48] detected some small amount of O_2_ from that reaction or the aluminum oxychloride formation reaction, with this theory behind their observation. The same theory may be responsible for Gaballah and coworkers’ results [35], where some reactivity and product formation were observed from Cr_2_O_3_ chlorination with Cl_2_ even though the yield was low.

**Figure 4:**
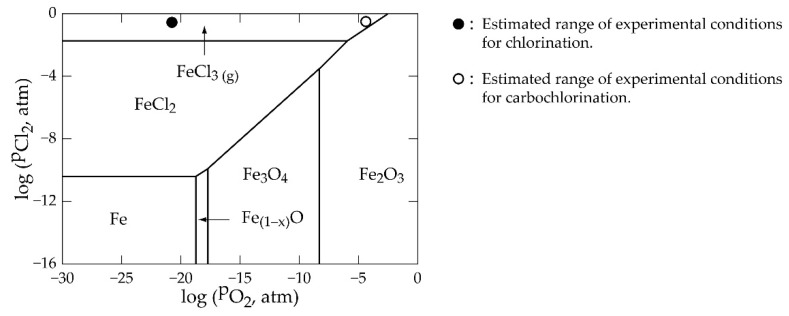
Phase stability diagram of Fe-O-Cl system at 800 °C.

The study also reveals that the +2 metal oxide reactions proceed to give products if chlorinated in gaseous chlorine, whilst those of higher valency do not. Contrary to the chlorine and carbon monoxide gas mixture, all oxides in this study are found capable of forming their respective metal chlorides. Inasmuch as the reducing gas (CO) enhances the chloride formation of almost all the oxides, it is coupled with the release of the greenhouse gas (CO_2_), which is a global concern. Iron oxide in the concentrate (as wustite or hematite) may produce either ferric or ferrous chloride. The Fe-O-Cl phase stability diagram (Figure 4) generated from the software confirms the two chlorides with a wider stability window for the ferrous form than the ferric form when the partial pressure of chlorine in the system is low.

However, ferric chloride may possibly be more stable than the ferrous form due to the predominancy of its production from reactions (Equations (5), (6), (16) and (17)) compared to ferrous chloride (Equations (4) and (15)) alongside the possibility of oxidation of the ferrous chloride to the ferric chloride. Reactions of wustite and hematite in the sample, which yield either hematite or magnetite in their products (as in Equation (8)), were mostly found to have strong thermodynamic tendencies (extremely low ${\Delta G}^{o}$). Hematite and/or magnetite are therefore expected to be predominant in an iron-rich cassiterite ore or concentrate. Kanari and co-workers’ results [49] should be a confirmation to this suspicion, where hematite and magnetite were confirmed as products in their study. The reaction of hematite, unlike wustite, was found not capable of yielding products in the chlorine gas alone except in the presence of CO. In spite of the software’s inability to give insight into the kinetics of reactions, the rate of the reactions is suspected to be more favorable in carbochlorination than chlorination, considering the significantly lowered ${\Delta G}^{o}$ values for the carbochlorination reactions. In both cases, the metal chlorides formed are predominantly in the gaseous phase, except for calcium and magnesium oxides, whose synthesized chlorides may be in the liquid phase. The formation of liquid calcium and magnesium chloride may passivate the sample and prevent diffusion of the gaseous reactants from accessing new reaction sites for the reaction to proceed. In other words, high quantities of calcium and magnesium oxide gangue in cassiterite concentrate are deleterious to valuable metal extraction by chlorine metallurgy with gaseous reactants.

As mentioned earlier, reactions of the higher oxidation state metal oxides in the concentrate (Al_2_O_3_, SiO_2_, TiO_2_, and SnO_2_) are all from the thermodynamic investigation and not possible in chlorine gas alone. It is observed in the literature that the preparation of the chlorides of these metals is predominantly through a reductive chlorination approach using chlorine gas and carbon or carbon monoxide as the reactive, confirming thermodynamic findings [45,50,51,52,53]. Carbochlorination of the oxides is known to be self-sustaining through its appreciably high exothermic reactions once commenced, but additional heat may be generated by the addition of a limited amount of oxygen to react with the reductant [45,52]. In spite of the identified feasible reductive chlorination reaction of SiO_2_, Hudon and Filippou [45] indicated that this gangue is inert to chlorine and, hence, a significant portion of it will remain in the residue.

## 3. Chlorination of Metal Oxides in a Cassiterite Concentrate with CaCl_2_ and MgCl_2_

In an attempt to curb the challenges linked to gaseous reactive compounds, solid reagents (i.e., CaCl_2_ and MgCl_2_) were investigated to explore their thermodynamic ability to replace gaseous mixtures for treating cassiterite concentrates. The reactions considered are expressed in Equations (25)–(38) below:

1/2SnO_2(s)_ + CaCl_2(s,l)_ → 1/2SnCl_4(g)_ + CaO_(s)_ (25)

MgO_(s)_ + CaCl_2(s,l)_ → MgCl_2(s,l)_ + CaO_(s)_ (26)

FeO_(s)_ + CaCl_2(s,l)_ → FeCl_2(s,l,g)_ + CaO_(s)_ (27)

1/3Fe_2_O_3(s)_ + CaCl_2((s,l)_ → 2/3FeCl_3(s,l,g)_ + CaO_(s)_ (28)

1/3Al_2_O_3(s)_ + CaCl_2(s,l)_ → 2/3AlCl_3(s,g)_ + CaO_(s)_ (29)

1/2TiO_2(s)_ + CaCl_2(s,l)_ → 1/2TiCl_4(g)_ + CaO_(s)_ (30)

1/2SiO_2_(s) + CaCl_2(s,l)_ → 1/2SiCl_4(g)_ + CaO_(s)_ (31)

1/2SnO_2(s)_ + MgCl_2(s,l)_ → 1/2SnCl_4(g)_ + MgO_(s)_ (32)

CaO_(s)_ + MgCl_2(s,l)_ → CaCl_2(s,l)_ + MgO_(s)_ (33)

FeO_(s)_ + MgCl_2(s,l)_ → FeCl_2(s,l,g)_ + MgO_(s)_ (34)

1/3Fe_2_O_3(s)_ + MgCl_2(s,l)_ → 2/3FeCl_3(s,l,g)_ + MgO_(s)_ (35)

1/3Al_2_O_3(s)_ + MgCl_2(s,l)_ → 2/3AlCl_3(s,g)_ + MgO_(s)_ (36)

1/2TiO_2(s)_ + MgCl_2(s,l)_ → 1/2TiCl_4(g)_ + MgO_(s)_ (37)

1/2SiO_2(s)_ + MgCl_2(s,l)_ → 1/2SiCl_4(g)_ + MgO_(s)_ (38)

**Figure 5:**
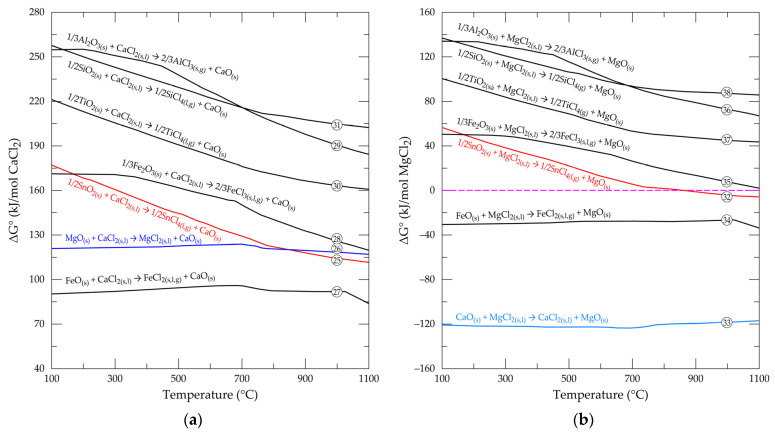
Evolution of standard free energy changes as function of temperature for the reactions of selected oxides with (**a**) CaCl_2_ and (**b**) MgCl_2_.

Figure 5a,b is the standard Gibbs free energy changes versus temperature for the reaction of the major oxides with calcium and magnesium chloride. Whilst calcium chloride shows no feasibility with any of the oxides, magnesium chloride, on the other hand, promises to produce chlorides for few of the oxides, particularly the lower oxidation state metal oxides (FeO and CaO). Their reactions have strong thermodynamic tendencies throughout the investigated temperature range. Cassiterite (SnO_2_) feasibility starts from 900 °C and above, which implies a possibility of extracting tin from the concentrate of cassiterite as tin chloride using magnesium chloride.

**Figure 6:**
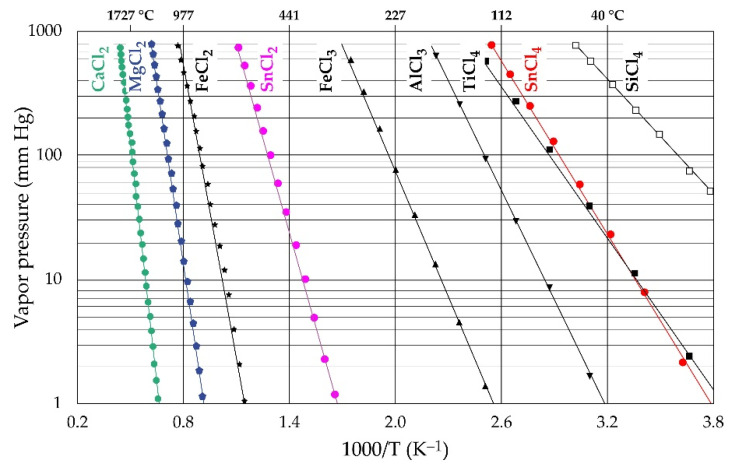
Evolution of vapor pressure of several chlorides as a function of temperature.

Figure 6 shows the variation of vapor pressure (VP) of the various chlorides expected in this study as a function of temperature. The diagram gives useful information for separating and recovering generated chlorides from the concentrate or upgrading it. From right to left of the figure is the increasing temperature dependence for the volatilization and, hence, the separation sequence. Thus, the separation is in the order SiCl_4_, SnCl_4_, TiCl_4_, AlCl_3_, FeCl_3_, SnCl_2_, FeCl_2_, MgCl_2_, and CaCl_2_ with increasing temperature. SiCl_4_ has the highest vapor pressure, suggesting that it may be the first to be recovered during the carbo-selective chlorination process. Elsewhere [45,47], SiO_2_, especially the α-polymorph, is said to be immune to the carbochlorination process regardless of thermodynamic findings. It is said that instead of synthesizing SiCl_4_ from SiO_2_ for easy volatilization from the chlorination material owing to its envisaged high VP, it rather remains and accumulates in the residue and thus in the chlorination equipment [45,47]. Concentrates or samples with high SiO_2_ composition have therefore been flagged as not suitable for some chlorination processes due to the inevitable frequent purging of the stuck SiO_2_ from the reactor [45,47]. The carbo-selective chlorination processes of this study find the observation otherwise due to the feasible SiO_2_ reaction observed in the study. The high VP of SnCl_4_ at the low temperature as well as the possible synthesis, from thermodynamic perspectives (Figure 3b and Figure 6), suggests a recovery route for tin from cassiterite concentrate. It is observed that there is the possibility for tin to form both stannic and stannous chloride. Despite this observation, there is a higher chance of achieving stannic chloride than stannous chloride in this study, due to the oxidizing nature of chlorine.

Tin extraction and recovery from cassiterite by this technique seems very suitable when magnesium chloride is employed as the chlorine source. This is because Figure 5b reveals that just a handful of the oxides (wustite and calcium oxide) show the possibility of chlorinating alongside SnO_2_ compared to the high chlorination tendencies of all oxides under carbochlorination. Purification and recovery of SnCl_4_ synthesized with MgCl_2_ from the mineral is thus expected to be easier compared to carbochlorination, which yields a spectrum of chloride mixtures. Nonetheless, CaCl_2_ and FeCl_2_, which are synthesized alongside SnCl_4_ in Figure 5b, are mostly liquids of low volatility, which remain in the system. In addition to passivating the active mineral surface and limiting the progress of reactions, as mentioned earlier, these liquefied chlorides can cause agglomeration of the sample and, hence, sintering [45,47]. Cassiterite concentrates used for tin extraction must thus, ideally, have a low CaO and FeO composition to circumvent this challenge. In cases where ferric rather than ferrous chloride is formed and/or the ferrous chloride is converted to the ferric chloride, it may be easily separated from the system owing to its lower VP than FeCl_2_. AlCl_3_, TiCl_4_, and SnCl_4_ have their VP close to each other, especially SnCl_4_ and TiCl_4_, such that TiCl_4_ will be the major contaminant of SnCl_4_ amongst the chlorides encountered.

## 4. Effect of SiO_2_ on Metal Oxide Chlorination in Cassiterite Concentrate

**Figure 7:**
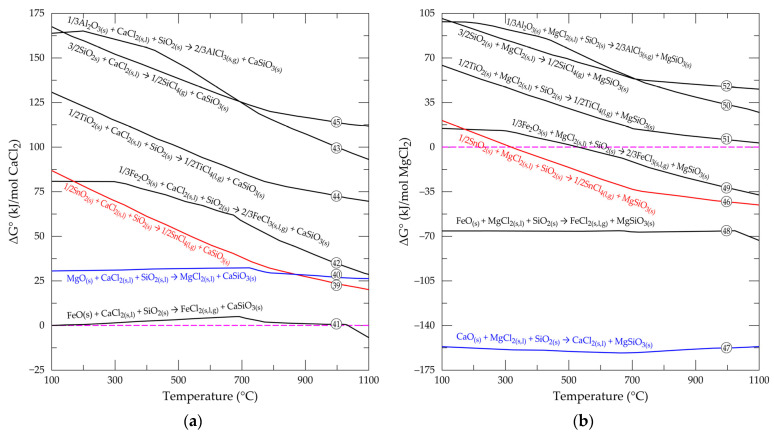
Evolution of standard free energy changes as function of temperature for the reactions of selected oxides with (**a**) CaCl_2_ and (**b**) MgCl_2_ in presence of SiO_2_.

Contrary to the negative effect of SiO_2_ to the process in the presence of gaseous reactive, as indicated by some researchers, chlorine from solid sources, upon breaking down to release its chlorine content, forms stable phases with the respective metal oxides and gangue (including SiO_2_) in the sample, as seen in Equations (39)–(52) [54]. The standard Gibbs free energy changes versus temperature for the resulting reaction are displayed in Figure 7. Earlier studies [55] have confirmed the positive influence of SiO_2_ on the synthesis of tin chloride whilst using calcium chloride and carbon to extract tin from the mineral. The carbon used was indicated as having a significant influence on the success of the study, but a potential contamination of the sample and increased operation cost to the process is suspect. Based on these limitations, the influence of quartz on the thermodynamic viability of tin chloride formation is investigated in the absence of carbon. The presence of this gangue is observed to have a greater decreasing effect on ${\Delta G}^{o}$ values when calcium chloride is used (Figure 7a) than in its absence (Figure 5a), though not enough to make the reactions feasible except in the presence of magnesium chloride (Figure 7b).

1/2SnO_2(s)_ + CaCl_2(s,l)_ + SiO_2(s)_ → 1/2SnCl_4(g)_ + CaSiO_3(s)_ (39)

MgO_(s)_ + CaCl_2(s,l)_ + SiO_2(s)_ → MgCl_2(s,l)_ + CaSiO_3(s)_ (40)

FeO_(s)_ + CaCl_2(s,l)_ + SiO_2(s)_ → FeCl_2(s,l,g)_ + CaSiO_3(s)_ (41)

1/3Fe_2_O_3(s)_ + CaCl_2(s,l)_ + SiO_2(s)_ → 2/3FeCl_3(s,l,g)_ + CaSiO_3(s)_ (42)

1/3Al_2_O_3(s)_ + CaCl_2(s,l)_ + SiO_2(s)_ → 2/3AlCl_3(s,g)_ + CaSiO_3(s)_ (43)

1/2TiO_2(s)_ + CaCl_2(s,l)_ + SiO_2(s)_ → 1/2TiCl_4(g)_ + CaSiO_3(s)_ (44)

3/2SiO_2(s)_ + CaCl_2(s,l)_ → 1/2SiCl_4(g)_ + CaSiO_3(s)_ (45)

1/2SnO_2(s)_ + MgCl_2(s,l)_ + SiO_2(s)_ → 1/2SnCl_4(g)_ + MgSiO_3(s)_ (46)

CaO_(s)_ + MgCl_2(s,l)_ + SiO_2(s)_ → CaCl_2(s,l)_ + MgSiO_3(s)_ (47)

FeO_(s)_ + MgCl_2(s,l)_ + SiO_2(s)_ → FeCl_2(s,l,g)_ + MgSiO_3(s)_ (48)

1/3Fe_2_O_3(s)_ + MgCl_2(s,l)_ + SiO_2(s)_ → 2/3FeCl_3(s,l,g)_ + MgSiO_3(s)_ (49)

1/3Al_2_O_3(s)_ + MgCl_2(s,l)_ + SiO_2(s)_ → 2/3AlCl_3(s,g)_ + MgSiO_3(s)_ (50)

1/2TiO_2(s)_ + MgCl_2(s,l)_ + SiO_2(s)_ → 1/2TiCl_4(g)_ + MgSiO_3(s)_ (51)

3/2SiO_2(s)_ + MgCl_2(s,l)_ → 1/2SiCl_4(g)_ + MgSiO_3(s)_ (52)

Similarly, ${\Delta G}^{o}$ values are found to decrease such that, cassiterite reaction starts occurring at 300 °C and beyond (Figure 7b), which is lower than the temperature it will start in the absence of SiO_2_ (Figure 5b). Hematite is the only new phase whose reaction is found to be feasible when the SiO_2_ is added to the thermochemical investigation.

**Figure 8:**
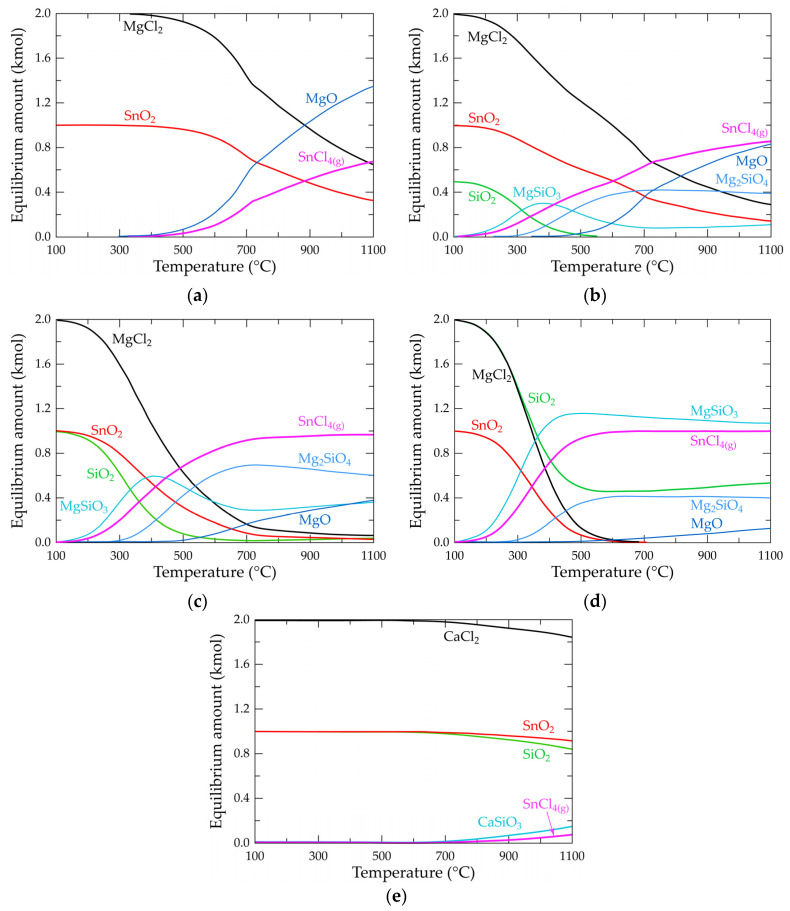
Evolution of the equilibrium composition as a function of temperature for systems: (**a**) Sn-Mg-Si-O-Cl in absence of SiO_2_; (**b**) Sn-Mg-Si-O-Cl at 0.5 kmol SiO_2_; (**c**) Sn-Mg-Si-O-Cl at 1.0 kmol SiO_2_; (**d**) Sn-Mg-Si-O-Cl at 2.0 kmol SiO_2_; and (**e**) Sn-Ca-Si-O-Cl at 1.0 kmol SiO_2_.

These revelations make chlorination with magnesium chloride in the presence of SiO_2_ interesting for the extraction and recovery of tin from cassiterite. It could also be used as an upgrading route for the niobium and tantalum pentoxides, which are usually associated with the mineral. Modeling of the process for further insight by using the equilibrium model of the software is shown in Figure 8.

The positive effect of tin extraction by the addition of SiO_2_ is confirmed by the modeling, where a decreased temperature for the formation of SnCl_4_ is observed. The study indicates, therefore, that the presence of SiO_2_, unlike that of other gangue, does not negatively influence all dry chlorination processes, as may be said by others. It has its own contribution to processes of this kind, with no limitation on the quantity required, as observed in this study. In Figure 8d, where an excess of the SiO_2_ was added, there was no observed limitation on extraction except for a reduced amount of the mineral that can be available in the feed, hence reducing the process efficiency. This finding must be born in mind during mineral processing of the ore to concentrate the mineral. That is, extreme beneficiation with the aim of rejecting SiO_2_,which, in effect, may result in the loss of some amount of cassiterite as well as critical and strategic minerals in the ore and must cautiously be considered if this extractive path will be followed. The modeling again confirms an earlier speculation that the formation of some stable phases from metal oxides and some gangue minerals (SiO_2_ in this study) support and drive the reaction in the forward direction. In Figure 8b–d (where SiO_2_ is present), a decreased temperature for tin extraction due to the formation of stable magnesium silicate phases is observed. The modeled Sn-Ca-Si-O-Cl equilibrium composition diagram is shown in Figure 8e, with a characteristic small amount of stannous chloride and minor calcium silicate, which confirms the unlikeliness for Equations (39)–(52) to proceed to the right.

## 5. Performance and Comparison of Chlorine Sources for Tin Extraction and Recovery from Cassiterite

**Figure 9:**
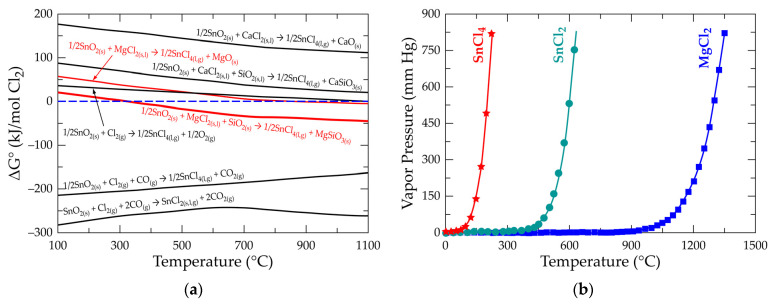
Thermodynamic deduction for the possible selective extraction of tin from its low-grade bearing materials: (**a**) comparison of the standard free energy changes as function of temperature for the reactions of SnO_2_ with selected chlorination agents; and (**b**) evolution of the vapor pressure of tin and magnesium chlorides as a function temperature.

Chlorine metallurgy owes its success partly to the source of chlorine, the mineral and metal to be treated, and the associated gangue. After exploring the behavior of major gangues, usually in association with cassiterite, and their impact on tin and other critical metal extraction and recovery processes, Figure 9a compares the performance of chlorine sources discussed against their thermodynamic tendencies to extract the metal from SnO_2_. Gaseous chlorine alone is found inefficient to extract tin from the mineral except in the presence of a reductant, in which case a stannic or stannous chloride is produced. The form of the metal produced is a function of the quantity of reducing agent, i.e., a high-reducing gaseous mixture may produce stannous chloride, whilst a low-to-moderate mixture produces the stannic counterpart. The Cl_2_/CO ratio may therefore be used as a preliminary determinant for the chloride form of metal to be synthesized. In cases where a mixture of both is suspected, Figure 9b (their VP evolution versus temperature) may be useful for their separation. SnCl_4_ vaporizes and can be recovered at lower temperatures (below 300 °C), whilst SnCl_2_ remains in its contained vessel. Amongst the chlorine salts studied, magnesium chloride seems promising for extracting the metal.

## 6. Conclusions

This study throws light on the thermodynamic assessment of chlorination using reagents from both gaseous and solid sources, with or without a reducing agent. Chlorine gas (Cl_2_) and Cl_2_ + CO are the gaseous chlorine reagents, whilst CaCl_2_ and MgCl_2_ are the solid chlorinating reagents considered. With direct chlorination with chlorine gas, upgrading of the concentrate could have been possible through the separation of calcium, magnesium, and iron oxides as their chlorides; however, the low VPs of these chlorides do not allow for their separation from the concentrate and subsequent upgrade. Carbochlorination studies, as seen with the Cl_2_ + CO mixture, may not qualify as a technique for upgrading the concentrate, since all oxides contained in the material have high thermodynamic tendencies to form chlorides, most of which have high VPs at different temperatures. Using a mixture of chlorine and carbon monoxide gas can thus ideally be suited for a selective recovery of the individual metal chlorides rather than upgrading. Considering the solid sources, MgCl_2_ is a promising salt that shows the thermodynamic possibility to recover the valuable metal tin as the chloride for downstream processing whilst leaving the majority of the gangue in residue. The extraction and recovery of tin from the concentrate with the salt may have an inherent advantage of upgrading its critical and strategic metal (particularly Nb and Ta) content for further processing. There is the possibility of a Cl_2_ + CO gas mixture to generate and separate Nb and Ta chlorides from the concentrate; nonetheless, it becomes complicated due to the complexity of the resulting gas mixture; more so, the low concentrations of Nb and Ta in the mixture will make their separation difficult. A stepwise extraction of tin from the concentrate with MgCl_2_, followed by the recovery of Nb and Ta from the resulting residue is recommended for achieving a wholistic extraction of valuable metals and associated critical and strategic metals from the cassiterite concentrate. This study forms the basis for the choice of MgCl_2_ for the selective extraction of tin from cassiterite concentrate prior to extraction of Nb and Ta obtained in the resulting residue, which will be presented in subsequent papers. Future studies may look at the thermodynamic view of the reaction of MgCl_2_ for the extraction of Nb and Ta that will be contained in the residue after the extraction of tin.

## Figures and Tables

**Table 1:** Composition of cassiterite (wt.%) in the concentrate or ore.

Oxide	Concentrate 1	Concentrate 2	Ore 1	Ore 2
SnO_2_	75.85	73.21	46.94	76.01
SiO_2_	8.85	7.39	4.95	5.93
TiO_2_	3.54	1.03	20.65	1.33
Fe_2_O_3_	2.75	5.53	17.79	7.41
Al_2_O_3_	1.88	0.63	2.42	3.19
MgO	1.85	_	_	0.13
CaO	0.81	3.35	0.81	_
Na_2_O	0.77	_	_	_
K_2_O	0.26	2.72	0.18	0.29
MnO	0.10	0.27	1.47	0.25
P_2_O_5_	0.09	_	0.54	0.40
Ta_2_O_5_	_	0.71	1.56	0.81
Nb_2_O_5_	_	0.59	1.43	0.62
Others	3.25	4.57	1.26	3.63

## Data Availability

No new data were created or analyzed in this study.
